# Specialized Care Resources for Diagnosis and Management of Patients Who Have Suffered Falls: Results of a National Survey in Geriatric Units

**DOI:** 10.3390/ijerph20115975

**Published:** 2023-05-27

**Authors:** Irene Bartolomé Martín, Ainhoa Esteve Arríen, Marta Neira Álvarez, Giovanna Cristofori, Bernardo Abel Cedeno-Veloz, Mariano Esbrí Víctor, Bárbara Pérez Pena, Alfonso González Ramírez, María Ángeles Caballero-Mora, on behalf of the Falls Study Group of Sociedad Española de Medicina Geriátrica (SEMEG)

**Affiliations:** 1 Servicio de Geriatría, Hospital Universitario de Guadalajara,19002 Guadalajara, Spain; ibartolome@sescam.jccm.es; 2 Geriatric Medicine, Universidad Castilla La Mancha, 45600 Toledo, Spain; ainhoa.esteve@uclm.es; 3 Geriatrician Department, Hospital Universitario Infanta Leonor, Universidad Complutense, 28031 Madrid, Spain; marta.neira@salud.madrid.org; 4 Geriatrician Department, Hospital Universitario Central de la Cruz Roja, 28003 Madrid, Spain; giovanna.cristofori@salud.madrid.org; 5 Geriatric Department, University Hospital of Navarre (HUN), 31008 Pamplona, Spain; ba.cedeno.veloz@navarra.es; 6 Geriatrician Department, Hospital Perpetuo Socorro Albacete, 02006 Albacete, Spain; mesbriv@gmail.com; 7 Geriatrician Department, Hospital Universitario Marqués de Valdecilla Santander, 39008 Santander, Spain; barbara.perez@scsalud.es; 8 Geriatrician Department, Hospital Universitario de Salamanca, 37007 Salamanca, Spain; alfonso.grsm@gmail.com; 9 Geriatrician Department, Hospital General Universitario de Ciudad Real, 13005 Madrid, Spain

**Keywords:** older people, falls, falls clinic, healthcare resources, comprehensive geriatric assessment

## Abstract

Introduction: Clinical guidelines recommend comprehensive multifactorial assessment and intervention to prevent falls and fractures in older populations. Methods: A descriptive study was conducted by the Falls Study Group of the Spanish Geriatric Medicine Society (SEMEG) to outline which types of healthcare-specific resources were assigned for fall assessment in Spanish geriatric departments. A self-reported seven-item questionnaire was delivered from February 2019 to February 2020. Where geriatric medicine departments were not available, we tried to contact geriatricians working in those areas. Results: Information was obtained regarding 91 participant centers from 15 autonomous communities, 35.1% being from Catalonia and 20.8% from Madrid. A total of 21.6% reported a multidisciplinary falls unit, half of them in geriatric day hospitals. Half of them reported fall assessment as part of a general geriatric assessment in general geriatric outpatient clinics (49.5%) and, in 74.7% of cases, the assessment was based on functional tests. A total of 18.7% reported the use of biomechanical tools, such as posturography, gait-rides or accelerometers, for gait and balance analysis, and 5.5% used dual X-ray absorptiometry. A total of 34% reported research activity focused on falls or related areas. Regarding intervention strategies, 59% reported in-hospital exercise programs focused on gait and balance improvement and 79% were aware of community programs or the pathways to refer patients to these resources. Conclusions: This study provides a necessary starting point for a future deep analysis. Although this study was carried out in Spain, it highlights the need to improve public health in the field of fall prevention, as well as the need, when implementing public health measures, to verify that these measures are implemented homogeneously throughout the territory. Therefore, although this analysis was at the local level, it could be useful for other countries to reproduce the model.

## 1. Introduction

Falls are a major problem of enormous magnitude among older adults. The high prevalence of falls is estimated at around 35% among those over 75 years old who live in a community [1]. The consequences of falls range from injuries in soft tissues to fractures, functional impairment, fear of falling syndrome, loss of quality of life and institutionalization. Complications are present in up to 50% of cases [1,2]. According to the National Survey of Hospital Morbidity published in February 2020, there were 58,467 discharges in Spain due to femur fractures among people over 75 years old in 2018. However, there is no unified record of falls.

Moreover, falls are a marker of frailty in older adults [3]. Their presence is related to a high number of underlying, underdiagnosed clinical problems (e.g., cognitive disorders, malnutrition, polypharmacy or functional decline) requiring specific assessment and intervention. In this context, the American Geriatrics Society and the British Geriatrics Society published in 2001—and updated in 2011—good clinical practice guidelines on falls among the elderly. This guide recommends the multidisciplinary assessment of all those older adults who have suffered a fall with consequences or two or more falls in the last year and includes the main aspects of such assessment and intervention [4,5]. Recently, international guidelines have been published along the same lines [6]. There is strong evidence that multidisciplinary intervention reduces the risk of falls by 23–31% [7,8,9] and the incidence of hip fractures by approximately 28% [10]. Consequently, fall and fracture prevention units have established themselves as the most efficient structures to deal with the growing incidence of falls and fractures in the older population, and the literature recommends wider implementation [11,12,13,14,15,16]. 

Finally, the geriatric resources available in the Spanish national territory show enormous dispersion and heterogeneity since geriatrics has been developed in different manners in the various Spanish political regions, which are called autonomous communities. Given that there is sufficient evidence of the benefits of assessment and intervention for falls, it seems necessary to create a map of available resources to address this problem in Spanish territory.

Therefore, the objective of the Grupo de Estudio de Caídas de la Sociedad Española de Medicina Geriátrica (SEMEG) is to describe the specific resources currently available for assessment and intervention in relation to patients who have suffered falls in geriatric services or units in the Spanish national territory.

## 2. Materials and Methods

The SEMEG Falls Study Group conducted a descriptive study to outline the availability of resources for the diagnosis and management of patients who have suffered falls in Spain. For this, a self-administered questionnaire consisting of seven questions was produced in electronic format (Appendix A) and sent to the Spanish geriatric services. In those areas where geriatric departments were not present, getriatricians working in the region were contacted to give feedback. The survey was sent to 200 centers. All survey responses were collected between February 2019 and February 2020, and frequency analysis was performed using SPSS version 22, USA. The survey design respected ethical principles, and participants were informed that personal data gathered in the survey would be treated as confidential and kept in automated data files authorized according to the Spanish law on personal data protection.

## 3. Results

**Figure 1:**
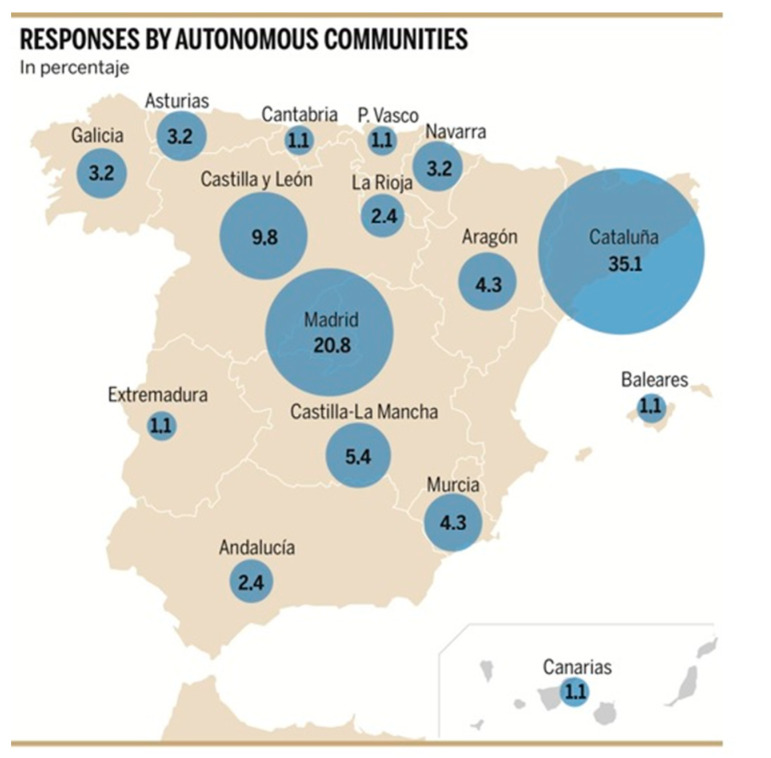
Own elaboration. Number of responses (as percentages) from autonomous communities.

There were 91 responses to the electronic form, corresponding to 15 of the 17 autonomous communities. The highest percentage was obtained from Catalonia (35.1%), followed by Madrid (20.8%). The complete details for the data can be seen in the Figure 1.

Resources available for fall assessment vary from one healthcare provider to another. Fall assessment can be carried out in a general outpatient clinic with or without a specific fall assessment protocol, in a multidisciplinary fall assessment unit that may or may not be located in a geriatric day hospital, or through a dedicated consultation focused only on the evaluation of falls (Table 1).

Regarding the tools and methods available for the evaluation of falls, 74.7% of geriatricians exclusively used functional performance tests, and 18.7% used other specific resources, such as posturography or technological devices for gait analysis, including accelerometers and electronic gait corridors. Additionally, 5.5% of those surveyed stated that they usually assessed bone and muscle health using densitometry (DEXA). Only in one of the cases was a dynamometer used alone to measure hand grip strength; however, in this case, other functional tests were not performed (Table 2).

Regarding the number of patients evaluated per month, more than half of the geriatricians reported that they usually attended to more than 10 patients per month and almost a quarter (18.7%) evaluated more than 20 patients per month (Table 3).

Regarding scientific activity, 65.9% of those surveyed did not carry out any type of study or research activity related to falls. A total of 28 geriatric services/units did report research activity, and 14 of them explained their areas of interest (frailty and sarcopenia for 35% and osteoporosis and fractures for 21.4%).

In terms of the physical exercise intervention programs available in their centers, in 40.7% of the cases, there were no physical exercise programs for rehabilitation of gait or falls. In one case (1.2%), there was no exercise program in the center but exercise was prescribed to be undertaken at home with regulated protocols. Among the 58.1% of services that had an exercise program, 11% were integrated with multidisciplinary falls units, 17.6% were integrated with geriatric daycare hospitals that were not specific to falls and 29.7% were not integrated with any of these care levels but were specific to falls (Table 4). The geriatric service carried out the exercise program only in two of the hospitals that had this resource and answered this question. The rest were run by rehabilitation services. 

Only 7.7% of the services surveyed (7) reported the number of patients participating in the exercise program monthly, and there were fewer than 11 patients in 71.4% of the cases (Table 5).

Finally, respondents were asked about exercise programs at the community level and the recommended programs established in the consultation to be carried out at home. Regarding the healthcare providers’ knowledge about exercise programs, 19.8% did not know what kinds of resources were available in their area. In 12.1% of the cases, they knew and used specific programs available for falls, and in 1.1% of the cases, the resource was known but not the way to refer patients to it. In addition, 30.8% answered that they did not have an established exercise protocol for this type of patient, instead giving general recommendations, and 16.5% prescribed exercise at home with already known guidelines (Table 6). 

## 4. Discussion

This study describes the results of a voluntary notification survey that aimed to determine the current geriatric resources for fall assessment and intervention in a particular territory. This was the first survey that brought out this question in Spain, and this work gives information for further studies on fall resources. Regarding the size of the sample, data were not obtained from all territories, as no information about the Valencian Community or the Autonomous Cities of Ceuta and Melilla was acquired, and we found that some communities were underrepresented.

Half of the participants reported that old people who had suffered falls were evaluated in the general geriatric outpatient clinic without a specific protocol for fall assessment. Several publications have stated recommendations about specific fall evaluations, patient referrals and the benefit of using specific protocols and tools to measure gait, improving the sensitivity of diagnoses and results [12,17]. Therefore, it seems necessary to reflect on what actions can be taken to improve routine clinical practice and to give that information to the health policymakers. In contrast, almost a quarter of those surveyed had multidisciplinary falls units, which are recognized as the best resource for approaches involving fall assessment and treatment [18]. However, it is possible that these data were overestimated since it is very likely that participation in the survey was higher among those units with greater interest in falls and, consequently, with more specific resources for them.

Regarding the protocols and tools available for assessing falls, almost three quarters of those who responded to the survey evaluated only with functional performance tests, such as the Short Physical Performance Battery, without using other tools, such as posturography, or technological devices, such as electronic gait corridors or accelerometers. Although some authors suggest that using these new technologies does not improve information or clinical results related to fall evaluation [18], they provide accurate information on gait patterns, speed, length and width and static and dynamic balance behavior under controlled conditions [19,20]. Posturography collects parameters relating to stability, postural control, center of gravity and balance, so its use is more than desirable in the assessment of elderly people who suffer falls. Even so, in the same way as technological devices for the biomechanical analysis of gait, no research work has found it to be essential in fall assessment. This suggests that it would be desirable to establish consensual recommendations on evaluations and tools to use in clinical practice. 

More than half of participants in the survey did not carry out any type of scientific activity related to falls. The World Health Organization recognizes falls as a major public health problem and recommends the implementation of comprehensive prevention strategies, prioritizing research and public health initiatives that create safer environments and reduce risk factors [21]. It considers it essential to promote the training of health professionals in preventive strategies based on scientific data and individual and community education on fall prevention [21]. For these reasons, it seems desirable to consider falls as a priority for geriatricians and other professionals who work with this target. In addition, it is also a great opportunity, given that research projects at the national, European and international levels prioritize those issues related to active and healthy aging.

Regarding interventions, in almost half of the cases, participants did not have their own physical exercise program to implement in their own center. Physical exercise is probably the most tested intervention in fall prevention [22,23,24]. It has been proven that it reduces the risk of falls both among institutionalized people and older people living in the community [4,5]. The exercise program that seems to reduce the risk the most overall is multicomponent exercise (cardiovascular endurance, flexibility, strength and balance) [22,24,25,26], although there are many studies that have analyzed training only in strength or cardiovascular endurance with good results [23,27]. It has been shown that exercise becomes more effective when it is carried out under professional direct control and not at home [26,28], and benefits are greater when the exercise is carried out in a group, since the subject’s confidence and motivation increase [5,22,29]. Taking the above into account, it is mandatory to have programs to implement or refer patients to in order to reduce the incidence and complications of falls.

Similarly, more than half of the participants in the survey reported being unaware of the existence of community physical exercise programs to refer people who have suffered falls to. Previous research has shown that the physical gain obtained after a physical exercise program is lost if it is not maintained over time [28,29,30,31,32], so if the hospital physical exercise program is not continued in the community, the benefit is lost after a period of time. Despite the fact that the published clinical guidelines do not endorse physical exercise carried out individually, the recent literature has begun to show that the prescription of individual programs of exercise at home with established protocols, such as the Vivifrail method [33], could be a good alternative in cases where it is not possible to refer the patient to a community group program. This prescription of individualized exercise as one more part of the patient’s treatment was reflected in this work, where one fifth of respondents who prescribed exercise programs specifically targeted their patients based on the benefit shown by the available evidence. Although fit people may have easier access to a community exercise program, it would be desirable to have hospital resources for those patients whose gait disorder or frailty management is more complex [34].

This study has the strength of being a first approximation of a map of current resources in Spain focused on assessment and intervention in older fallers. The geographical differences and lack of homogeneity in resources highlight the need for standardization and improvement in this area. These findings give us the opportunity to initiate intervention plans in the different areas evaluated: falls units, evaluation standards and intervention programs with exercise at the hospital and community levels.

We also consider that, although the study was carried out in Spain, it highlights the need to improve public health in terms of fall prevention and implement public health measures to verify that these measures are implemented homogeneously throughout the territory evaluated. Therefore, although this analysis was undertaken at the local level, it could be useful for other countries to reproduce the model.

The study had limitations. The survey design only allowed for a descriptive analysis; comparing fall prevention activity in relation to geographical resources needs more data related to the department organization and patient follow-up. Less than 50% of the centers responded to the survey and some geographical areas had low representation due to geriatrics not being extensively developed in the healthcare system. Finally, closed-ended survey questions could have increased the frequency of missing answers. To the best of our knowledge, no other studies have been conducted to describe fall assessment resources in other countries. These limitations should be considered when explaining results and planning future research in this area.

## 5. Conclusions

Finally, according to the results of the study, we found that most resources related to falls are concentrated in two autonomous communities, and patients are generally evaluated in general outpatient clinics without specific fall protocols and mainly with functional tests exclusively. Most of the centers do not have any physical exercise programs designed for falls and they give general recommendations. Finally, there is an opportunity to improve the implementation of specific resources for fall assessment and intervention care. 

## Figures and Tables

**Table 1:** Resources for assessment and diagnosis of falls.

Resources	Frequency	Percentage
A consultation dedicated to the evaluation of falls	5	5.5
A general outpatient clinic (not dedicated to falls) with a specific fall assessment protocol	14	15.4
A general geriatric outpatient clinic with comprehensive geriatric assessment of geriatric syndromes	45	49.5
A multidisciplinary fall assessment unit located in a geriatric day hospital	11	12.1
A multidisciplinary fall assessment unit not located in a geriatric day hospital	13	14.3
No answer	3	3.3
Total	83	100

**Table 2:** Fall assessment tools.

Fall Assessment Tools	Frequency	Percentage
Posturograph, device for gait evaluation, functional tests and dynamometer	5	5.5
Posturograph without device for gait evaluation or functional tests	5	5.5
Functional test and gait evaluation device	2	2.2
Evaluation with functional tests only	68	74.7
Grip strength assessment with dynamometer only	1	1.2
No answer	5	5.5
Total	91	100

**Table 3:** Number of patients evaluated per month.

Number of Patients/Month	Frequency	Percentage
1–5	23	25.3
6–10	19	20.9
11–20	29	31.0
>20	17	18.7
No answer	3	3.3
Total	91	100

**Table 4:** Intervention programs available with physical exercise at the hospital level.

Intervention	Frequency	Percentage
Multidisciplinary fall assessment unit with physical exercise program	10	11
Exercise or rehabilitation program in geriatric day hospital but not designed for falls	16	17.6
Fall-specific exercise program performed in the hospital but not part of the falls unit	27	29.7
No rehabilitation/exercise program for gait or falls	36	39.6
Prescription of exercise at home with already known protocols or guidelines (such as those of Vivifrail)	1	1.1
No answer	1	1.1
Total	91	100

**Table 5:** Number of people participating in the exercise programs each month.

Number of People Participating in the Exercise Programs Each Month	Frequency	Percentage	Percentage without an Answer
5–10	5	5.5	71.4
11–15	1	1.1	14.3
>16	1	1.1	14.3
No answer	84	92.3	
Total	91	100	100

**Table 6:** Healthcare providers’ knowledge about physical exercise programs at the community level.

Providers’ Knowledge about Exercise Programs	Frequency	Percentage
I do not know what resources are available at the community level in my area	18	19.8
There is a specific fall or gait intervention program that I can refer my patients to	11	12.1
There are resources in my area but I do not know how I can refer patients from the hospital	1	1.1
There are resources that provide specific exercise programs that are not specific to falls or walking	16	17.6
We do not have an established protocol and general recommendations are given	28	30.8
We prescribe exercise at home with already known protocols or guidelines (such as those of Vivifrail)	15	16.5
No answer	2	2.2
Total	91	100

## Data Availability

The authors publish in this article all data available of the survey.

## Appendix A. Survey

1. Regarding the assessment and diagnosis of falls. Please indicate only one of the following options, as the most valid in the practice of your unit or service:

A. A multidisciplinary fall assessment unit located in a Geriatric Day Hospital. If so, indicate who is the person responsible for this unit

B. A multidisciplinary fall assessment unit not located in the Geriatric Day Hospital. If so, indicate who is the person responsible for this unit

C. A monographic consultation on the evaluation of falls. Indicate if there is a person responsible for this query

D. A general outpatient clinic (not monographic on falls) with a specific fall assessment protocol

E. A general geriatric outpatient clinic with comprehensive geriatric assessment of geriatric syndromes

2. Regarding the tools for assessing the falls in your Service. Please tick all the valid options:

A. There is a posturographer

B. There is a gait evaluation system such as gait rite, accelerometers or others.

C. Gait assessment is performed with functional performance tests such as SPPB, gait speed, or the Timed up and Go test

D. Strength is assessed with dynamometers

E. Quantitative assessment of the muscle is performed with bioimpedancemetry..

F. Quantitative assessment is performed with DEXA.

3. How many patients per month are evaluated in this consultation?:

A. 1–5/month

B. 5–10/month

C. 10–20/month

D. >20 /month

4. Do you carry out any scientific activity in relation to falls in your unit/service? If so, could you indicate the line of investigation?

A. Yes

B. No

5. Would someone in your service be interested in collaborating in multicenter studies on falls?

A. Yes

B. No

6. Regarding intervention programs with physical exercise at the hospital level. Which of the following options is more valid in the practice of your unit or service:

A. There is a multidisciplinary fall assessment unit with a physical exercise program. If so, indicate approximately how many people can participate in the program on a monthly basis

B. There is an exercise or rehabilitation program at the Geriatric Day Hospital, but it is not designed for falls or gait rehabilitation

C. There is a specific exercise program for falls and gait that is carried out in the hospital but is not part of the falls unit. If so, indicate if it is developed by Rehabilitation or another service and which one

D. They do not have any rehabilitation/exercise program for gait or falls.

7. Regarding intervention programs with physical exercise at the community level. Which of the following options is closest to your usual practice (several can be indicated):

A. I do not know what resources are available at the community level in my área

B. There is a specific fall or gait intervention program that I can refer my patients to. If so, indicate place and person responsible

C. There are resources in my area but I don’t know how I can refer patients from the hospital

D. There are resources where exercise programs are carried out in general, although they are not specific for falls or walking. If so, indicate if possible which are these centers

E. We prescribe exercise at home with already known protocols or recommendation guidelines (such as those of vivifrail). Please indicate some of them

F. We do not have an established protocol and general recommendations are given.

## References

[1] Tinetti M.E., Speechley M., Ginter S.F. (1988). Risk Factors for Falls Among Elderly Persons Living in the Community. N. Engl. J. Med..

[2] Pérez-Ros P., Martínez-Arnau F.M., Tarazona-Santabalbina F.J. (2019). Risk Factors and Number of Falls as Determinants of Quality of Life of Community-Dwelling Older Adults. J. Geriatr. Phys. Ther..

[3] Fried L.P., Tangen C.M., Walston J., Newman A.B., Hirsch C., Gottdiener J., Seeman T., Tracy R., Kop W.J., Burke G. (2001). Cardiovascular Health Study Collaborative Research Group. Frailty in Older Adults: Evidence for a Phenotype. J. Gerontol. A Biol. Sci. Med. Sci..

[4] (2001). Guideline for the prevention of falls in older persons. American Geriatrics Society, British Geriatrics Society, and American Academy of Orthopaedic Surgeons Panel on Falls Prevention. J. Am. Geriatr. Soc..

[5] Panel on Prevention of Falls in Older Persons, American Geriatrics Society, British Geriatrics Society (2011). Summary of the Updated American Geriatrics Society/British Geriatrics Society Clinical Practice Guideline for Prevention of Falls in Older Persons. J. Am. Geriatr. Soc..

[6] Montero-Odasso M., Van der Velde N., Martin F.C., Petrovic M., Tan M.P., Ryg J., Aguilar-Navarro S., Alexander N.B., Becker C., Blain H. (2022). World Guidelines for Falls Prevention and Management for Older Adults: A Global Initiative. Age Ageing.

[7] DiBardino D., Cohen E.R., Didwania A. (2012). meta-Analysis: Multidisciplinary Fall Prevention Strategies in the Acute Care Inpatient Population. J. Hosp. Med..

[8] Gillespie L.D., Robertson M.C., Gillespie W.J., Sherrington C., Gates S., Clemson L.M., Lamb S.E. (2012). Interventions for Preventing Falls in Older People Living in the Community. Cochrane Database Syst. Rev..

[9] Preventing Falls and Harm From Falls in Older People. Best Practice Guidelines for Australian Community Care. https://www.safetyandquality.gov.au/publications-and-resources/resource-library/preventing-falls-and-harm-falls-older-people-guidebook-australian-community-care.

[10] Alcalde Tirado P. (2010). Miedo a Caerse [Fear of falling]. Rev. Esp. Geriatr. Gerontol..

[11] Dent E., Lien C., Lim W.S., Wong W.C., Wong C.H., Ng T.P., Woo J., Dong B., De la Vega S., Hua Poi P.J. (2017). The Asia-Pacific Clinical Practice Guidelines for the Management of Frailty. J. Am. Med. Dir. Assoc..

[12] Falls: Assessment and Prevention of Falls in Older People. www.nice.org.uk/guidance/CG161.

[13] Gomez F., Curcio C.L., Brennan-Olsen S.L., Boersma D., Phu S., Vogrin S., Suriyaarachchi P., Duque G. (2019). Effects of the Falls and Fractures Clinic as an Integrated Multidisciplinary Model of Care in Australia: A Pre-Post Study. BMJ Open.

[14] Cameron I.D., Dyer S.M., Panagoda C.E., Murray G.R., Hill K.D., Cumming R.G., Kerse N. (2018). Interventions for Preventing Falls in Older People in Care Facilities and Hospitals. Cochrane Database Syst. Rev..

[15] Perell K.L., Manzano M.L., Weaver R., Fiuzat M., Voss-McCarthy M., Opava-Rutter D., Castle S.C. (2006). Outcomes of a Consult Fall Prevention Screening Clinic. Am. J. Phys. Med. Rehabil..

[16] Thomas S., Miller M., Whitehead C., Crotty M. (2010). Falls Clinics: An Opportunity To Address Frailty and Improve Health Outcomes (Preliminary Evidence). Aging Clin. Exp. Res..

[17] 2018 NICE Impact Report on Falls and Fragility Fractures. https://www.bgs.org.uk/resources/2018-nice-impact-report-on-falls-and-fragility-fractures..

[18] Baker R. (2006). Gait Analysis Methods in Rehabilitation. J. Neuroeng. Rehabil..

[19] Graham J.E., Ostir G.V., Kuo Y.F., Fisher S.R., Ottenbacher K.J. (2008). Relationship between Test Methodology and Mean Velocity in Timed Walk Tests: A Review. Arch. Phys. Med. Rehabil..

[20] Graham J.E., Ostir G.V., Fisher S.R., Ottenbacher K.J. (2008). Assessing Walking Speed in Clinical Research: A Systematic Review. J. Eval. Clin. Pract..

[21] Falls. http://www.who.int/mediacentre/factsheets/fs344/es/.2018.

[22] Casas Herrero Á., Cadore E.L., Martínez Velilla N., Izquierdo Redin M. (2015). Physical Exercise in the Frail Elderly: An Update. Rev. Esp. Geriatr. Gerontol..

[23] Lopez P., Pinto R.S., Radaelli R., Rech A., Grazioli R., Izquierdo M., Cadore E.L. (2018). Benefits of Resistance Training in Physically Frail Elderly: A Systematic Review. Aging Clin. Exp. Res..

[24] Lopez P., Izquierdo M., Radaelli R., Sbruzzi G., Grazioli R., Pinto R.S., Cadore E.L. (2018). Effectiveness of Multimodal Training on Functional Capacity in Frail Older People: A Meta-Analysis of Randomized Controlled Trials. J. Aging Phys. Act..

[25] Viladrosa M., Casanova C., Ghiorghies A.C., Jürschik P. (2017). Effectiveness of Physical Exercise on Fitness in Frail Older Adults: A Systematic Review of Randomised Trials. Rev. Esp. Geriatr. Gerontol..

[26] García-Molina R., Ruíz-Grao M.C., Noguerón-García A., Martínez-Reig M., Esbrí-Víctor M., Izquierdo M., Abizanda P. (2018). Benefits of a Multicomponent Falls Unit-Based Exercise Program in Older Adults With Falls in Real Life. Exp. Gerontol..

[27] Ramirez-Campillo R., Diaz D., Martinez-Salazar C., Valdés-Badilla P., Delgado-Floody P., Méndez-Rebolledo G., Cañas-Jamet R., Cristi-Montero C., García-Hermoso A., Celis-Morales C. (2016). Effects of Different Doses of High-Speed Resistance Training on Physical Performance and Quality of Life in Older Women: A Ran-Domized Controlled Trial. Clin. Interv. Aging..

[28] Toraman N.F., Ayceman N. (2005). Effects of Six Weeks of Detraining on Retention of Functional Fitness of Old People After Nine Weeks of Multicomponent Training. Br. J. Sports Med..

[29] Falls in Older People: Assessing Risk and Prevention. https://www.nice.org.uk/guidance/cg161.

[30] Vogler C.M., Menant J.C., Sherrington C., Ogle S.J., Lord S.R. (2012). Evidence of Detraining After 12-Week Home-Based Exercise Programs Designed To Reduce Fall-Risk Factors in Older People Recently Discharged From Hospital. Arch. Phys. Med. Rehabil..

[31] Eggenberger P., Theill N., Holenstein S., Schumacher V., De Bruin E.D. (2015). Multicomponent Physical Exercise With Simultaneous Cognitive Training To Enhance Dual-Task Walking of Older Adults: A Secondary Analysis of a 6-Month Randomized Controlled Trial With 1-Year Follow-Up. Clin. Interv. Aging.

[32] Tarazona-Santabalbina F.J., Gómez-Cabrera M.C., Pérez-Ros P., Martínez-Arnau F.M., Cabo H., Tsaparas K., Salvador-Pascual A., Rodriguez-Mañas L., Viña J.A. (2016). Multicomponent Exercise Intervention That Reverses Frailty and Improves Cognition, Emotion, and Social Networking in the Community-Dwelling Frail Elderly: A Randomized Clinical Trial. J. Am. Med. Dir. Assoc..

[33] Casas-Herrero Á., Sáez de Asteasu M.L., Antón-Rodrigo I., Sánchez-Sánchez J.L., Montero-Odasso M., Marín-Epelde I., Ramón-Espinoza F., Zambom-Ferraresi F., Petidier-Torregrosa R., Elexpuru-Estomba J. (2022). Effects of Vivifrail Multicomponent Intervention on Functional Capacity: A Multicentre, Randomized Controlled Trial. J. Cachexia Sarcopenia Muscle.

[34] Estrategia De Promoción de la Salud y Prevención en el SNS. Actualización Del Documento de Consenso Sobre Prevención de la Fragilidad en la Persona Mayor (2022). https://www.sanidad.gob.es/ciudadanos/pdf/Estrategia_de_Salud_Publica_2022.pdf.

[35] Casas Anguita J., Repullo Labrador J.R., Donaldo Campos J. (2003). La encuesta como técnica de investigación. Elaboración de cuestionarios y tratamiento estadístico de los datos. Aten. Primaria.

